# Nonparametric Tests for Exponentiality Against IFRA Alternatives Based on Cumulative Extropy Measures

**DOI:** 10.3390/e28020208

**Published:** 2026-02-11

**Authors:** Anfal A. Alqefari

**Affiliations:** Department of Statistics and Operations Research, College of Science, Qassim University, P.O. Box 6644, Buraydah 51482, Saudi Arabia; aa.alqefari@qu.edu.sa; Tel.: +966-541461447

**Keywords:** nonparametric tests, exponentiality testing, increasing failure rate average (IFRA), cumulative residual extropy, cumulative past extropy, entropy, failure analysis, simulation, 62N05, 62G10, 62G20, 94A17

## Abstract

This paper develops two nonparametric test statistics for testing exponentiality against alternatives in the increasing failure rate average (IFRA) class. The proposed procedures are constructed using information-theoretic functionals, namely the cumulative residual extropy and the cumulative past extropy of the first-order statistic. Exploiting fundamental properties of IFRA distributions, we derive explicit inequality relations that motivate the test statistics and establish their asymptotic normality under mild regularity conditions. To facilitate practical implementation, scale-invariant versions of the proposed tests are introduced, ensuring that their limiting distributions do not depend on unknown scale parameters. A comprehensive Monte Carlo simulation study demonstrates that the proposed tests possess strong power properties and frequently outperform several established competitors, particularly for moderate to large sample sizes. The applicability and effectiveness of the methodology are further illustrated through analyses of real lifetime datasets arising in reliability studies. The proposed tests are shown to be particularly effective for moderate sample sizes and provide a competitive alternative to existing IFRA-based procedures.

## 1. Introduction

Statistical distribution theory provides the foundation for modeling, inference, and hypothesis testing in reliability analysis and survival studies. In this context, verifying distributional assumptions for lifetime data is a central problem with direct methodological and practical implications. Among the various aging classes, the IFRA class occupies a prominent position, as it constitutes the smallest class of lifetime distributions that contains the exponential model and remains closed under the formation of coherent systems. This structural property is essential in system reliability analysis and makes the IFRA class attractive for both theoretical investigations and applied reliability modeling.

Let X denote a non-negative random variable representing a lifetime, with cumulative distribution function (cdf) F(x)=P(X≤x) and survival function $\bar{F}(x)=P(X>x)$. The distribution F is said to belong to the IFRA class if the function $(-log\;\bar{F}(x))/x$ is increasing in x>0, or equivalently if and only if

(1) $$\bar{F}(bx)\geq [\bar{F}(x){]}^{b},x>0,for\;all\;0<b<1.$$

Equality in (1) holds if and only if F is the exponential distribution. A comprehensive treatment of IFRA distributions and their fundamental properties can be found in Barlow and Proschan [1].

Methods for quantifying uncertainty associated with probability distributions are deeply rooted in information theory. Among the most influential measures in this field is Shannon’s differential entropy, introduced by Shannon [2] as a fundamental quantifier of uncertainty. For a non-negative random variable X with probability density function (pdf) f, the Shannon differential entropy is defined as H(X)=E[−log f(X)], where log(⋅) means natural logarithms, provided that the expectation exists. While Shannon entropy is a cornerstone measure of uncertainty, a recent complementary measure known as extropy was introduced by Lad et al. [3]. Extropy serves as a dual to Shannon entropy, offering a distinct yet related quantification of uncertainty that is particularly useful in reliability and information-theoretic analyses. The duality between entropy and extropy can be understood through their respective roles in measuring the surprise or information content associated with a probability distribution. For a non-negative random variable X with pdf f and cdf F, the extropy is defined as:

(2) $$J\left(X\right)=-\frac{1}{2}\int_{0}^{\infty }\;\;f^{2}\left(x\right)dx.$$

Unlike Shannon entropy, which measures the average log-density of a distribution, extropy measures the concentration of the square of the density. Extropy exhibits several advantageous properties in statistical inference, particularly in connection with proper scoring rules. Specifically, it is intrinsically linked to the total logarithmic scoring rule, a fundamental tool for evaluating predictive distributions see Agro et al. [4], Capotorti et al. [5], Gneiting and Raftery [6], among others. This connection makes extropy-based measures particularly suitable for hypothesis testing and model selection. Differential entropy quantifies the disparity of a probability density function *f*(*x*) from the uniform distribution, reflecting uncertainty in the distribution’s shape. Extropy, introduced as entropy’s complementary dual, similarly measures deviation from uniformity but through a distinct functional form. Despite both capturing aspects of dispersion and uncertainty, they exhibit fundamental differences from some substantial and subtle differences; differential entropy H(X) ranges over [−∞,∞], whereas extropy J(X) is confined to [−∞,0). Moreover, H(X)<J(X) due to the fact that $2x\log x<x^{2}$ for all *x *> 0.

In the analysis of lifetime data, uncertainty measures expressed in terms of probability density functions may be less suitable in nonparametric frameworks, where densities are difficult to estimate accurately. In contrast, cumulative distribution and survival functions arise naturally in lifetime modeling and are typically easier to estimate and interpret. This has motivated the development of cumulative versions of entropy- and extropy-based measures. Along these lines, Jahanshahi et al. [7] introduced the cumulative residual extropy (CREX) by replacing the density function in (2) with the survival function

$$EJ\left(X\right)=-\frac{1}{2}\int_{0}^{\infty }\;\;{\bar{F}}^{2}\left(x\right)dx,$$

yielding a natural measure of uncertainty associated with the remaining lifetime. From a dual perspective, Tahmasebi and Toomaj [8] proposed the cumulative past extropy (CPEX), as

$$\bar{ℇ}J\left(X\right)=-\frac{1}{2}\int_{0}^{\infty }\;\;(1-F^{2}\left(x\right))dx,$$

which employs the cumulative distribution function to quantify uncertainty related to past lifetimes. Due to their cumulative formulation and direct reliance on distribution functions which are easier to estimate nonparametrically, both CREX and CPEX provide a robust and interpretable foundation for building inferential procedures in reliability theory and aging analysis. This paper leverages these cumulative extropy measures to develop new nonparametric tests for exponentiality against IFRA alternatives.

In reliability engineering and survival analysis, distributions are often assumed to exhibit adverse aging, reflecting deterioration of system performance over time. A classical representation of such behavior is the increasing failure rate (IFR) property, for which nonparametric inference has been widely studied. Despite its appeal, the IFR assumption is often restrictive in practice, as it excludes heavy-tailed models and fails to accommodate bathtub-shaped hazard rates frequently observed in real data. Weaker aging notions consistent with adverse aging have therefore been introduced, among which the IFRA class represents a natural relaxation while preserving key structural features. A fundamental problem in reliability theory is to test whether a lifetime distribution is exponential against alternatives exhibiting aging behavior, such as IFR, IFRA, new better than used (NBU), and related classes. Since the exponential distribution represents the canonical no-aging benchmark, departures from it indicate deterioration or improvement effects with important implications in engineering, survival analysis, and quality control. Such testing problems naturally motivate the development of nonparametric procedures that exploit structural properties of IFRA distributions. Accordingly, in this paper we consider testing $H_{0}:F\left(x\right)=1-e^{-\lambda x},\;x>0,\;\lambda >0\;vs.\;H_{1}:F$ is IFRA.

The problem of testing exponentiality against aging alternatives has been extensively investigated. Proschan and Pyke [9] laid early theoretical foundations for testing monotone failure rates, while Hollander and Proschan [10] introduced influential total time on test (TTT)-based tests for the NBU class. Deshpande [11] proposed simple and effective tests for exponentiality against IFRA alternatives, and Aly and Lu [12] developed a unified asymptotic framework covering several aging classes. Further contributions by Ahmad [13,14] based on moment inequalities have enriched this literature. Building on these foundations, more recent work has refined and extended nonparametric tests for aging classes. For instance, Ahmad and Mugdadi [15] derived further moment-based inequalities, providing new test statistics for the IFRA, NBUC, and DMRL classes. Srivastava et al. [16] applied modern machine-learning frameworks from artificial intelligence to the problem of testing membership in the IFRA and NBU families. A significant line of inquiry into the more IFRA ordering was advanced by Izadi and Khaledi [17], who developed new statistical tests for this stronger aging property, with further theoretical and empirical results presented in their subsequent work [18]. These works collectively highlight the depth of nonparametric methodology developed for reliability and life-testing problems.

The remainder of the paper is organized as follows. Section 2 establishes the theoretical foundation of the proposed methodology by introducing the extropy-based deviation measures and rigorously deriving the fundamental inequality results induced by the IFRA class. Section 3 focuses on the inferential development of the proposed procedures, encompassing the derivation of their asymptotic properties, the construction of scale-invariant test statistics, and a thorough evaluation of finite-sample performance via extensive Monte Carlo simulation studies, complemented by illustrative real-data applications. Finally, Section 4 concludes the paper by summarizing the main theoretical and empirical findings and outlining several promising directions for future research. For simplicity, throughout the remainder of this paper, we write $g^{a}(x)$ instead of $[g(x){]}^{a}$. Unless stated otherwise, it is assumed that all expectations and integrals appearing in the paper exist and are finite. In addition, *n* denotes the order-statistic parameter, while *N* refers to the sample size.

## 2. Cumulative Extropy Inequalities

This section investigates fundamental properties of cumulative residual extropy and cumulative past extropy that are instrumental for developing the proposed testing procedures. Let $X_{1},X_{2},\cdots ,X_{n}$ be a random sample of size n from a distribution F. Denoted by $X_{1:n}=min\left\{X_{1},X_{2},\ldots ,X_{n}\right\}$ the first order statistic. The survival function of $X_{1:n}$ is given by

(3) $${\bar{F}}_{1:n}\left(x\right)={\bar{F}}^{n}\left(x\right),\;\;for\;x>0.$$

In the context of reliability engineering, $X_{1:n}$ represents the lifetime of a series system. It is well known that the IFRA property is preserved under series formation; that is, if F belongs to the IFRA class, then the distribution of $X_{1:n}$ is also IFRA. This preservation property will be exploited to derive several useful inequality results, which form the theoretical basis for the test statistics developed in the subsequent sections.

The proportional hazards (PH) model is one of the most widely used frameworks in survival analysis and reliability theory, with applications spanning reliability modeling, customer attrition analysis, preventive maintenance scheduling, and optimal replacement policies. Seminal contributions by Cox [19], Kobbacy et al. [20], Makis and Jardine [21], and Van den Poel and Larivière [22] have established the PH model as a fundamental tool in both theoretical and applied statistical analyses. Under the PH model, the survival function of a non-negative random variable $X^{a}$ is defined as ${\bar{F}}_{a}(x)={\bar{F}}^{a}(x)$ for x≥0 and a>0, where $\bar{F}(x)$ is the baseline survival function of X with finite mean μ=E(X), and a is the proportional hazard parameter.

The proportional reversed hazards (PRH) model, introduced by Gupta et al. [23], has received increasing attention in reliability theory and survival analysis. As the dual counterpart of the proportional hazards (PH) model, the PRH framework characterizes lifetime distributions through the reversed hazard rate. Specifically, under the PRH model, the cumulative distribution function of a non-negative random variable ${\tilde{X}}^{a}$ with cdf ${\tilde{F}}_{a}(x)=F^{a}(x)$ for x≥0 and a>0, where F(x) is the baseline cdf of X and a is the proportional reversed hazard parameter. Let $X_{1:n}^{b}$ and ${\tilde{X}}_{1:n}^{b}$ represent the PH and PRH models of $X_{1:n}$, respectively.

It is worth emphasizing that the PH and PRH models are not introduced here as stand-alone lifetime models, but rather as transformation frameworks for generating families of distributions that preserve IFRA properties. This perspective provides a clear theoretical justification for their inclusion and plays a central role in motivating and interpreting the cumulative extropy inequalities established in the subsequent analysis. Moreover, this transformation-based framework directly facilitates the construction of test statistics that exploit IFRA-preserving inequalities under systematic scaling.

The following results establish key properties of these models that will be used in the subsequent development.

**Theorem** **1.** *Let* X *be a non-negative random variable with cumulative distribution function* F *and survival function* $\bar{F}$ *. Assume that the CREX and cumulative past extropy of the corresponding first-order statistics exist and are finite. If* F *belongs to the IFRA class, then for all* 0<b<1

(i) $EJ\left(X_{1:n}\right)\leq bEJ(X_{1:n}^{b})$,
(ii) $\bar{E}J(X_{1:n})\leq b\bar{E}J({\tilde{X}}_{1:n}^{b})$.

**Proof.** 
(i) Since *F* belongs to the IFRA class, relation (1) implies that the survival function of the first-order statistic $X_{1:n}$ satisfies:

(4) $${\bar{F}}_{1:n}\left(bx\right)\geq {\bar{F}}_{1:n}^{b}\left(x\right),x>0,for\;all\;0<b<1.$$

Squaring both sides, integrating over (0, ∞), and using Equation (3), we obtain:

$$\begin{array}{cc} EJ\left(X_{1:n}^{b}\right) & \;=-0.5\int_{0}^{\infty }\;\;{\bar{F}}_{1:n}^{2b}(x)dx\\ & \;\geq -0.5\int_{0}^{\infty }\;\;{\bar{F}}_{1:n}^{2}(bx)dx\\ & \;=-0.5b^{-1}\int_{0}^{\infty }\;\;{\bar{F}}_{1:n}^{2}(u)du\;(\;\mathrm{with}\;u=bx)\\ & \;=b^{-1}EJ\left(X_{1:n}\right),for\;all\;0<b<1. \end{array}$$

(ii) Let $F_{1:n}(bx)=1-{\bar{F}}_{1:n}(bx)$, x>0, 0<b<1. Relation (4) implies $F_{1:n}(bx)\leq 1-{\bar{F}}_{1:n}^{b}(x)$, which gives $\left(1-F_{1:n}^{2}(bx)\right)\geq 1-{\left[1-{\bar{F}}_{1:n}^{b}(x)\right]}^{2}$ for x>0 and 0<b<1. Integrating both sides of this relation and using Equation (4), we obtain

$$\begin{array}{cc} \bar{E}J\left({\tilde{X}}_{1:n}^{b}\right) & =-0.5\int_{0}^{\infty }\;\;(1-{\left[1-{\bar{F}}_{1:n}^{b}(x)\right]}^{2})dx\\ & \geq -0.5\int_{0}^{\infty }\;\;\left(1-F_{1:n}^{2}(bx)\right)dx\\ & =-0.5b^{-1}\int_{0}^{\infty }\;\;\left(1-F_{1:n}^{2}(u)\right)du\;(\mathrm{where}\;u=bx)\\ & =b^{-1}\bar{E}J\left(X_{1:n}\right),\;for\;all\;0<b<1, \end{array}$$

which completes the proof. □

It is important to note that the inequalities established in Theorem 1 are tight under the null hypothesis of exponentiality. Consequently, any systematic deviation from equality provides a natural signed measure of departure from exponentiality toward IFRA alternatives. This observation motivates the construction of the following deviation measures. The class of tests developed in this paper for detecting departures from exponentiality against IFRA alternatives is firmly rooted in the inequality framework established in Theorem 1. These inequalities involve an integer-valued tuning parameter n, which governs the behavior of the first-order statistic and, consequently, influences the power and sensitivity of the resulting tests. Practical considerations and guidelines for selecting appropriate values of n are discussed to ensure a balance between theoretical robustness and empirical effectiveness. As a direct and illustrative application of Theorem 1, we now present the following example, which demonstrates the construction of the proposed extropy-based test statistics.

**Example** **1.** *Assume that* $X_{1},X_{2},\cdots ,X_{n}$ *constitute a random sample of size* n *drawn from a common Weibull distribution with survival function *

(5) $$\bar{F}\left(x\right)=e^{-\lambda x^{\beta }},x>0,\lambda >0,\beta >0.$$

*It follows that* ${\bar{F}}_{1:n}^{b}(x)=e^{-nb\lambda x^{\beta }}$ for x>0. *From Theorem 1(i), for* λ>0 *and* β>0 *we have*

$$bEJ\left(X_{1:n}^{b}\right)=-\frac{b}{2}\int_{0}^{\infty }\;{\bar{F}}_{1:n}^{2b}\left(x\right)dx=-b\Gamma \left(\frac{1}{\beta }\right)/\beta (nb\lambda {)}^{\frac{1}{\beta }},$$

*and*

$$EJ\left(X_{1:n}\right)=-\frac{1}{2}\int_{0}^{\infty }\;{\bar{F}}_{1:n}^{2}\left(x\right)dx=-\Gamma \left(\frac{1}{\beta }\right)/\beta (n\lambda {)}^{\frac{1}{\beta }}.$$

*It follows directly from these representations that* $EJ\left(X_{1:n}\right)\leq bEJ\left(X_{1:n}^{b}\right)$* for *0<b<1 *and* β≥1, *which is consistent with Part (i) of Theorem 1 since the Weibull distribution is IFRA when* β≥1*. Furthermore, we can derive the following representations:*

$$\begin{array}{cc} \bar{E}J\left({\tilde{X}}_{1:n}^{b}\right) & =-\frac{1}{2}\int_{0}^{\infty }\;\;(1-{\left[1-{\bar{F}}_{1:n}^{b}(x)\right]}^{2})dx\\ & =-\frac{1}{2}\int_{0}^{\infty }\;\;\left(1-{\left[1-e^{-nb\lambda x^{\beta }}\right]}^{2}\right)dx=-\frac{b\left(2-2^{-\frac{1}{\beta }}\right)\Gamma \left(\frac{1}{\beta }\right)}{\beta (nb\lambda {)}^{\frac{1}{\beta }}}, \end{array}$$

*and*

$$\begin{array}{cc} \bar{E}J\left({\tilde{X}}_{1:n}\right) & =-\frac{1}{2}\int_{0}^{\infty }\;\;\left(1-F_{1:n}^{2}(x)\right)dx\\ & =-\frac{1}{2}\int_{0}^{\infty }\;\;\left(1-{\left[1-e^{-n\lambda x^{\beta }}\right]}^{2}\right)dx=-\frac{\left(2-2^{-\frac{1}{\beta }}\right)\Gamma \left(\frac{1}{\beta }\right)}{\beta (n\lambda {)}^{\frac{1}{\beta }}}, \end{array}$$

*where *λ>0* and *β>0*. Similar reasoning applies in this setting, leading us to conclude that*

$$\bar{E}J\left(X_{1:n}\right)\leq b\bar{E}J\left({\tilde{X}}_{1:n}^{b}\right),\;0<b<1,\;\beta \geq 1,$$

*as anticipated from Part (ii) of Theorem 1. Finally, equality holds in both parts when* β=1*, corresponding to the exponential distribution, which serves as the boundary case between IFRA and DFRA classes.*

## 3. Testing the IFRA Property

Testing exponentiality against IFRA alternatives occupies a central role in reliability engineering and survival analysis. The exponential distribution represents the canonical “no-aging” benchmark; rejecting it in favor of IFRA alternatives provides formal statistical evidence of adverse aging behavior, critical for maintenance scheduling, warranty analysis, and system design decisions. While numerous nonparametric tests for IFRA alternatives exist (e.g., Deshpande [11], Aly and Lu [12]), most rely on moment inequalities or TTT transforms, which may lack sensitivity to certain aging patterns or require complex asymptotic approximations.

The approach developed here offers a distinct information-theoretic perspective by leveraging cumulative extropy measures of the first-order statistic, we construct tests that directly quantify uncertainty reduction associated with aging. This framework provides three key advantages: (i) the test statistics arise naturally from fundamental IFRA-preserving inequalities (Theorem 1), yielding interpretable signed measures of departure from exponentiality; (ii) the cumulative formulation avoids density estimation, enhancing robustness in nonparametric settings; and (iii) the resulting procedures exhibit competitive power, particularly for moderate to large samples. This bridges information theory and reliability testing in a novel, practically useful way.

Let $X_{1},X_{2},\ldots ,X_{n}$ be a random sample from a continuous probability distribution with cdf F. Our objective is to develop nonparametric procedures for testing the null hypothesis

$$H_{0}:F(x)=1-e^{-\lambda x},x,\lambda >0,$$

with λ unspecified against the alternative $H_{1}$ that F belongs to the increasing failure rate average class, but is not exponential. Our test is motivated by considering Part (i) of Theorem 1 and the following integral as a measure of deviation, for a given F, from $H_{0}$ to $H_{1}$. Let

$$\begin{array}{cc} \delta_{n,b}^{1} & =bEJ\left(X_{1:n}^{b}\right)-EJ\left(X_{1:n}\right)\\ & =0.5[\int_{0}^{\infty }\;\;({\bar{F}}^{2n}(x)-b{\bar{F}}^{2nb}(x))dx],\;for\;all\;0<b<1. \end{array}$$

Integration by parts simplifies to

(6) $$\delta_{n,b}^{1}=\int_{0}^{\infty }\;\;xJ_{1}\left(F\left(x\right)\right)dF\left(x\right),$$

where $J_{1}(u)=n[(1-u{)}^{2n-1}-b^{2}(1-u{)}^{2nb-1}]$, for all 0<u<1. Under the null hypothesis $H_{0}$, i.e., the exponential distribution the measure $\delta_{n,b}^{1}$ is zero (see Example 1). Large values of the estimator for $\delta_{n,b}^{1}$ provide evidence against the null hypothesis.

The following lemma plays a key role in establishing the asymptotic normality of the estimator defined in (6).

**Lemma** **1.** *Assume that *$J_{1}(u)=n[(1-u{)}^{2n-1}-b^{2}(1-u{)}^{2nb-1}]$*, for all *0<u<1*. For a fixed *n≥1*, and all *0<b<1*, we have *$\left|J_{1}(u)\right|\leq 2n(1-u{)}^{2nb-1},\;$*for all * 0≤u≤1.

**Proof.** 
For 0<b<1 and 0<u<1, the triangle inequality yields

$$\left|J_{1}(u)\right|\leq n[(1-u{)}^{2n-1}+b^{2}(1-u{)}^{2nb-1}]\leq n\left[(1-u{)}^{2n-1}+(1-u{)}^{2nb-1}\right]$$

for all 0<u<1, where the second inequality follows from the fact that $b^{2}<1$ and $(1-u{)}^{2nb-1}>$ 0. Because 2n−1>2nb−1 and 0<1−u<1, it follows that $(1-u{)}^{2n-1}<(1-u{)}^{2nb-1}$. Therefore, for all 0<u<1,

$$n\left[(1-u{)}^{2n-1}+(1-u{)}^{2nb-1}\right]\leq n\left[(1-u{)}^{2nb-1}+(1-u{)}^{2nb-1}\right]=2n(1-u{)}^{2nb-1}$$

which completes the proof. □

Alternatively, another test is inspired by Part (ii) of Theorem 1 and is based on the following integral, which serves as a measure of deviation for a given F from $H_{0}$ to $H_{1}$:

$$\begin{array}{cc} \delta_{n,b}^{2} & =b\bar{E}J\left(X_{1:n}^{b}\right)-\bar{E}J\left(X_{1:n}\right)\\ & =0.5\left[\int_{0}^{\infty }\;\;\left(\left(1-F_{1:n}^{2}(x)\right)-b(1-{\left[1-{\bar{F}}_{1:n}^{b}(x)\right]}^{2})\right)dx\right],\;for\;all\;0<b<1. \end{array}$$

Like the previous measure, after applying integration by parts, it simplifies to:

(7) $$\delta_{n,b}^{2}=\int_{0}^{\infty }\;\;xJ_{2}\left(F\left(x\right)\right)dF\left(x\right),$$

where $J_{2}(u)=n[(1-u{)}^{n-1}-(1-u{)}^{2n-1}-b^{2}\left((1-u{)}^{nb-1}-(1-u{)}^{2nb-1}\right)]$, for all 0<u<1.

It is worth noting that the proposed deviation measures are signed by construction. Positive values of the statistics provide evidence in favor of IFRA-type aging, whereas negative values indicate departures from exponentiality toward DFRA-type alternatives. This sign interpretation will be used throughout the simulation and data analysis sections. The following lemma will be used to prove the asymptotic normality of the estimator (7).

**Lemma** **2.** *Assume that *$J_{2}(u)=n[\left(1-u{)}^{n-1}-(1-u{)}^{2n-1}-b^{2}((1-u{)}^{nb-1}-(1-u{)}^{2nb-1}\right)]$*, for all *0<u<1*. For a fixed *n≥1*, and all *0<b<1*, we have *$\left|J_{2}(u)\right|\leq 2n(1-u{)}^{nb-1},$ *for all* 0≤u≤1.

**Proof.** 
For 0<b<1 and 0<u<1, the triangle inequality gives

$$\begin{array}{cc} \left|J_{2}(u)\right| & \leq n\left|(1-u{)}^{n-1}-(1-u{)}^{2n-1}\right|+b^{2}\left|(1-u{)}^{nb-1}-(1-u{)}^{2nb-1}\right|\\ & \leq n\left[(1-u{)}^{n-1}+(1-u{)}^{nb-1}\right]. \end{array}$$

The second inequality follows from observing that $(1-u{)}^{n-1}-(1-u{)}^{2n-1}<(1-u{)}^{n-1}$, since $(1-u{)}^{2n-1}>0$, and similarly $(1-u{)}^{nb-1}-(1-u{)}^{2nb-1}<(1-u{)}^{nb-1}$ since $(1-u{)}^{2nb-1}>0$, for all 0<b<1 and n≥1. Moreover, $b^{2}<1$ for 0<b<1. Now, because nb−1<n−1, for 0<b<1 and 0<u<1, we have $\left|J_{2}(u)\right|\leq 2n(1-u{)}^{nb-1}$, for all 0<u<1, which completes the proof. □

Let $X_{1},X_{2},\ldots ,X_{N}$ be a sequence of independent and identically distributed (i.i.d.) continuous, non-negative random variables, with order statistics $X_{1:N}\leq X_{2:N}\leq \cdots \leq X_{N:N}$. The empirical distribution function corresponding to F(x) is given by ${\hat{F}}_{N}(x)=\frac{1}{N}\sum_{i=1}^{N}\;I_{\left(X_{i}\leq x\right)}$, which can be expressed as

$${\hat{F}}_{N}(x)=\left\{\begin{array}{l} 0,x<x_{1:N}\\ \frac{i}{N},x_{1:N}\leq x\leq x_{i+1:N},(i=1,2,\ldots ,N-1)\\ 1,x>x_{N:N} \end{array}\right.$$

where $I_{A}$ is the indicator function of event A. Substituting the empirical distribution function $F_{N}$ for F in (8), an estimator of the $\delta_{n,b}^{1}$ that utilizes a nonparametric approach, derived from the L-functional estimator, is given by:

$${\hat{\delta }}_{n,b}^{1}=\int_{0}^{\infty }\;xJ_{1}\left({\hat{F}}_{N}(x)\right)d{\hat{F}}_{N}(x)=\frac{1}{N}\sum_{i=1}^{N}\;J_{1}\left(\frac{i}{N}\right)X_{i:N}$$

where $J_{1}(u)=n[(1-u{)}^{2n-1}-b^{2}(1-u{)}^{2nb-1}]$, for all 0<u<1. Similar arguments can be applied to ${\hat{\delta }}_{n,b}^{2}$. The following theorem establishes the asymptotic normality of the test statistic ${\hat{\delta }}_{n,b}^{1}$.

**Theorem** **2.** *Let *X* be a continuous non-negative random variable with finite second moment *$E\left(X^{2}\right)<\infty$*. Let *$\delta_{n,b}^{1}$* be defined as in (8) and consider its empirical estimator *${\hat{\delta }}_{n,b}^{1}$*. Define*

(8) $$\sigma^{2}\left(J_{1},F\right)=\int_{0}^{\infty }\;\;\int_{0}^{\infty }\;\;(F\left(min\left(x,y\right)\right)-F\left(x\right)F\left(y\right))J_{1}(F\left(x\right))J_{1}(F\left(y\right))dx\;dy,$$

*where *$J_{1}(u)=n[(1-u{)}^{2n-1}-b^{2}(1-u{)}^{2nb-1}]$ *for* 0<u<1*. Then, as *$N\to \infty ,\sqrt{N}\left({\hat{\delta }}_{n,b}^{1}-\delta_{n,b}^{1}\right)$ *converges in distribution to a normal random variable with mean zero and variance* $\sigma^{2}\left(J_{1},F\right)$ *for all* nb≥1/4.

**Proof.** 
The estimator ${\hat{\delta }}_{n,b}^{1}=\frac{1}{N}\sum_{i=1}^{N}\;J_{1}\left(\frac{i}{N}\right)X_{i:N}$ is an L-statistic (linear function of order statistics) with the weight function $J_{1}(u)$ applied to the empirical quantile process. The smooth function $J_{1}(u)$ is bounded and continuous on the open interval (0, 1) when nb≥1/4 due to Lemma 1. Moreover, the assumption $E\left(X^{2}\right)<\infty$ is sufficient to satisfy the second-moment condition required for the convergence of the underlying empirical process. Given the above conditions, Theorems 2 and 3 of Stigler [24] directly imply that $\sqrt{N}\left({\hat{\delta }}_{n,b}^{1}-\delta_{n,b}^{1}\right)$ converges in distribution to a normal distribution with mean zero and finite variance given in (8). Under the alternative hypothesis (*F* is non-exponential and IFRA), the parameter $\delta_{n,b}^{1}$ is non-zero. The smoothness of $J_{1}(u)$ and the non-degeneracy of *F* ensure that $\sigma^{2}\left(J_{1},F\right)>0$, as N→∞ and this completes the proof. □

The distribution of ${\hat{\delta }}_{n,b}^{1}$ is not scale-invariant. To construct a test that is invariant under changes in scale, we normalize by the sample mean. Specifically, we define the scale-invariant test statistic

(9) $${\hat{\delta }}_{n,b}^{1^{⋆}}={\hat{\delta }}_{n,b}^{1}/\bar{X},$$

where $\bar{X}$ is the sample mean. This adjustment ensures that the distribution of ${\hat{\delta }}_{n,b}^{1^{⋆}}$ under $H_{0}$ does not depend on the unknown scale parameter, allowing us to calibrate the test using a fixed standard exponential. The limiting distribution of ${\hat{\delta }}_{n,b}^{1}$, derived using Theorem 2 and Slutsky’s theorem (see, e.g., Cramer [25]), is presented in the following theorem.

**Theorem** **3.** *Under the conditions of Theorem 2, it follows that*

$$\sqrt{N}\left({\hat{\delta }}_{n,b}^{1^{⋆}}-\frac{\delta_{n,b}^{1}}{E(X)}\right)\overset{D}{\to }N\left(0,\frac{\sigma^{2}\left(J_{1},F\right)}{E^{2}(X)}\right),\;\mathrm{for}\;\mathrm{all}\;nb\geq 1/4$$

*We now derive the limiting distribution under the null hypothesis of exponentiality. Since the statistic* ${\hat{\delta }}_{n,b}^{1^{⋆}}$ *is scale-invariant, without loss of generality, we may assume the null hypothesis as* $H_{0}:F(x)=1-e^{-x}$* for *x>0. *Under this choice, one finds that* $\delta_{n,b}^{1}=0$ *and* $E^{2}(X)=1$. *Consequently, the test statistic* $\sqrt{N}{\hat{\delta }}_{n,b}^{1^{⋆}}$ *is asymptotically* $N\left(0,\sigma^{2}\left(J_{1},F\right)\right)$. *Large values of* ${\hat{\delta }}_{n,b}^{1^{⋆}}$ *indicate an increasing failure rate on average, while small values* *suggest a* *decreasing failure rate. To address the dependence of (10) on the unknown distribution function, we use the consistent estimator defined by Jones and Zitikis* [26]

$${\hat{\sigma }}^{2}\left(J_{1},F_{N}\right)=\sum_{i=1}^{N-1}\;\sum_{j=1}^{N-1}\;\left(min\left(\frac{i}{N},\frac{j}{N}\right)-\frac{i}{N}\frac{j}{N}\right)J_{1}\left(\frac{i}{N}\right)J_{1}\left(\frac{j}{N}\right)\left(X_{i+1:N}-X_{1:N}\right)\left(X_{j+1:N}-X_{j:N}\right).$$

*The decision rule for rejecting* $H_{0}$ *in favor of* $H_{1}$ *at significance level* α *is:*

(10) $$\frac{\sqrt{2N}{\hat{\delta }}_{n,b}^{1^{⋆}}}{\hat{\sigma }\left(J_{1},F_{N}\right)}>z_{1-\alpha },$$

*where *$z_{1-\alpha }$* is the *(1−α)*-quantile of the standard normal distribution. We now present a similar result for the cumulative extropy of the first-order statistics. To this aim, we propose a non-parametric estimator of the CPEX, derived from the *L*-functional estimator, defined as:*

$${\hat{\delta }}_{n,b}^{2}=\int_{0}^{\infty }\;xJ_{2}\left({\hat{F}}_{N}\left(x\right)\right)d{\hat{F}}_{N}\left(x\right)=\frac{1}{N}\sum_{i=1}^{N}\;J_{2}\left(\frac{i}{N}\right)X_{i:N},$$

*where *$J_{2}(u)=n[(1-u{)}^{n-1}-(1-u{)}^{2n-1}-b^{2}\left((1-u{)}^{nb-1}-(1-u{)}^{2nb-1}\right)]$*, for all *0<u<1*. The asymptotic normality of the test statistic *${\hat{\delta }}_{n,b}^{2}$* is established in the following theorem, with the proof omitted due to its similarity to the proof of Theorem 2.*

**Theorem** **4.** *Assume that *$E\left(X^{2}\right)<\infty$* and let *$\delta_{n,b}^{2}$* be defined as in (7). Define*

(11) $$\sigma^{2}\left(J_{2},F\right)=\int_{0}^{\infty }\;\int_{0}^{\infty }\;\;(F\left(min\left(x,y\right)\right)-F\left(x\right)F\left(y\right))J_{2}(F\left(x\right))J_{2}(F\left(y\right))dx\;dy,$$

*where* $J_{2}(u)=n[(1-u{)}^{n-1}-(1-u{)}^{2n-1}-b^{2}\left((1-u{)}^{nb-1}-(1-u{)}^{2nb-1}\right)]$*, for all* 0<u<1*. Then, as* $N\to \infty ,\sqrt{N}\left({\hat{\delta }}_{n,b}^{2}-\delta_{n,b}^{2}\right)$ *converges in distribution to a normal random variable with mean zero and variance* $\sigma^{2}\left(J_{2},F\right)$ *for all* nb≥1/2.

**Proof.** 
The estimator ${\hat{\delta }}_{n,b}^{2}=\frac{1}{N}\sum_{i=1}^{N}\;J_{2}\left(\frac{i}{N}\right)X_{i:N}$ is an L-statistic with the weight function $J_{2}(u)$. Under the condition nb≥1/2, the smooth function $J_{2}(u)$ is bounded and continuous on the open interval (0, 1) due to Lemma 2. Combined with the moment condition $E\left(X^{2}\right)<\infty$, Theorems 2 and 3 of Stigler [24] imply that $\sqrt{N}\left({\hat{\delta }}_{n,b}^{2}-\delta_{n,b}^{2}\right)$ converges in distribution to a normal distribution with mean zero and finite variance $\sigma^{2}\left(J_{2},F\right)$. Under the alternative hypothesis, $\delta_{n,b}^{2}$ is non-zero and $\sigma^{2}\left(J_{2},F\right)>0$ is finite as N→∞, completing the proof. □

*Similar to* ${\hat{\delta }}_{n,b}^{1}$, *we define the scale-invariant test statistic as follows:*

(12) $${\hat{\delta }}_{n,b}^{2^{⋆}}=\frac{{\hat{\delta }}_{n,b}^{2}}{\bar{X}},\;for\;all\;0<b<1.$$

*This adjustment ensures that the distribution of* ${\hat{\delta }}_{n,b}^{2^{⋆}}$ *under* $H_{0}$ *does not depend on the unknown scale parameter, allowing us to calibrate the test using a fixed standard exponential. Similar to Theorem 3, the limiting distribution of* ${\hat{\delta }}_{n,b}^{2}$, *derived using Theorem 4 and Slutsky’s theorem is presented in the following theorem*.

**Theorem** **5.** *Under the conditions of Theorem 4, it follows that*

$$\sqrt{N}\left({\hat{\delta }}_{n,b}^{2^{⋆}}-\frac{\delta_{n,b}^{2}}{E(X)}\right)\overset{D\;}{\to }N\left(0,\frac{\sigma^{2}\left(J_{2},F\right)}{E^{2}(X)}\right),\;\mathrm{for}\;\mathrm{all}\;nb\geq 1/2.$$

*Moreover, we establish the asymptotic distribution of the test statistic* ${\hat{\delta }}_{n,b}^{2^{⋆}}$ *under the null hypothesis that the data arise from an exponential distribution. Since* ${\hat{\delta }}_{n,b}^{2^{⋆}}$ *is invariant under scale transformations, under* $H_{0}:F(x)=1-e^{-x},x>0$ *, the population parameter satisfies* $\delta_{n,b}^{2}=0$ *and* $E\left(X^{2}\right)=1$ *. It then follows that, under* $H_{0}$ *,* $\sqrt{N}{\hat{\delta }}_{n,b}^{2^{⋆}}\overset{D}{\to }N\left(0,\sigma^{2}\left(J_{2},F\right)\right)\;as\;N\to \infty .$

*Large positive values of* ${\hat{\delta }}_{n,b}^{2^{⋆}}$ *provide evidence of an IFRA alternative. A consistent estimator of the asymptotic variance is given by the form*

$${\hat{\sigma }}^{2}\left(J_{2},F_{N}\right)=\sum_{i=1}^{N-1}\;\sum_{j=1}^{N-1}\;\left(min\left(\frac{i}{N},\frac{j}{N}\right)-\frac{i}{N}\frac{j}{N}\right)J_{2}\left(\frac{i}{N}\right)J_{2}\left(\frac{j}{N}\right)\left(X_{i+1:N}-X_{1:N}\right)\left(X_{j+1:N}-X_{j:N}\right),$$

*Accordingly, at level-* α*, test rejects the null hypothesis of exponentiality in favor of *$H_{1}$* whenever*

(13) $$\frac{\sqrt{2N}{\hat{\delta }}_{n,b}^{2^{⋆}}}{\hat{\sigma }\left(J_{2},F_{N}\right)}>z_{1-\alpha },$$

*where* $z_{1-\alpha }$* is defined previously. In the next section, we assess the finite-sample power performance of the proposed test through an extensive Monte Carlo study and compare it against several well-established competitors.*

**Remark** **1.** *A parallel methodology can be developed for testing exponentiality against decreasing failure rate in average (DFRA) alternatives, the dual aging class. For large values of N, the null hypothesis of exponentiality is rejected in favor of a DFRA alternative, at level of significance α, whenever*

$$\frac{\sqrt{2N}{\hat{\delta }}_{n,b}^{1^{⋆}}}{\hat{\sigma }\left(J_{1},F_{N}\right)}<-z_{1-\alpha },\;and\;\;\;\frac{\sqrt{2N}{\hat{\delta }}_{n,b}^{2^{⋆}}}{\hat{\sigma }\left(J_{2},F_{N}\right)}<-z_{1-\alpha },$$

*where* $z_{1-\alpha }$ *is defined previously.*

### 3.1. Extension to Right-Censored Data

*Suppose the lifetime observations *$X_{1},\ldots ,X_{n}$* are subject to independent right censoring by non-negative random variables *$T_{1},\ldots ,T_{n}$* with continuous distribution function K, where each *$T_{i}$* is independent of *$X_{i}.$* The observable data then consist of the pairs *$\left\{(Z_{i},\delta_{i}):\;i=1,\ldots ,n\right\}$*, where *

$$Z_{i}=\min\left\{X_{i},T_{i}\right\},\;\;\;\delta_{i}=I\left\{X_{i}\leq \;T_{i}\right\},$$

*with *$\delta_{i}=1$* indicating an uncensored observation and *$\delta_{i}=0$* a censored one. A natural extension of the proposed methodology to this setting replaces the empirical distribution function with the Kaplan–Meier estimator (Kaplan and Meier [27])*

$$\hat{F}\left(t\right)=\sum_{i=1}^{N}{\hat{p}}_{i}I_{\left(0,t\right)}\left(Z_{i:N}\right)=1-\prod_{i:z_{i}\leq t}\left(1-\frac{\delta_{i}}{n-i+1}\right),$$

*where *$Z_{1:N}\leq \cdots \leq \;Z_{N:N}$* denote the ordered observed times, *${\hat{p}}_{i}=(\delta_{i}/(n-i+1))\;\prod_{j=1}^{i-1}\left(1-(\delta_{j}/(n-j+1))\right)$*, for *i=1,… , n* and *$\delta_{i}$* the corresponding censoring indicators*.

*Substituting *$\hat{F}$* for F in the definition of the first deviation measure yields the censored-data estimator *

$$\delta_{n,b}^{1}(\hat{F})=\sum_{i=1}^{N}\;{\hat{p}}_{i}J_{1}(\sum_{j=1}^{i}{\hat{p}}_{j})X_{i:N}.$$

*Under standard regularity conditions (independence of censoring, continuity of F and K, and positivity of the censoring survival function on the support of F), Theorem 4.1 of* [28] *implies that, as* N→∞*,* $\sqrt{N}\left(\delta_{n,b}^{1}(\hat{F})-\delta_{n,b}^{1}\right)$ *where * $J_{1}^{⋆}\left(u\right)=J_{1}\left(u\right)-\delta_{n,b}^{1}$* and the asymptotic variance is *

$$\sigma^{2}\left(J_{1}^{⋆},F\right)=2\int_{0}^{\infty }\;\;\int_{x}^{\infty }\;\;F\left(x\right)\bar{F}\left(y\right)J_{1}^{⋆}\left(F\left(x\right)\right)J_{1}^{⋆}\left(F\left(y\right)\right)\left(\int_{0}^{x}\frac{dF\left(s\right)}{\bar{F}\left(s\right)\bar{K}\left(s\right)}\right)dy\;dx,$$

*with *$\bar{K}(u)=1-K(u)$* denoting the survival function of the censoring times. Under the null hypothesis *$H_{0}$* of exponentiality, *$J_{1}^{⋆}\left(u\right)=J_{1}\left(u\right)$*. A consistent estimator *$\sigma^{2}(J_{1}^{⋆},\hat{F})$* is obtained by replacing F by *$\hat{F}$* and *$\bar{K}$* with the empirical censoring survival function*

$${\bar{K}}_{N}\left(t\right)=\left(\frac{1}{N}\right)\sum_{i=1}^{N}I\left(Z_{i}>t\right).$$

*In similar with the uncensored case, we reject *$H_{0}$* in favor of *$H_{1}$* at the significant level *α* when we have *$\frac{\sqrt{N}\delta_{n,b}^{1}(\hat{F})}{\hat{\sigma }\left(J_{1}^{⋆},\hat{F}\right)}>z_{1-\alpha }.$* An analogous construction applies to the second deviation measure *$\delta_{n,b}^{2}$* based on cumulative past extropy, yielding a second censored-data test statistic *$\delta_{n,b}^{2}(\hat{F})$* with identical asymptotic justification. This extension enables the practical application of the proposed methodology to survival data commonly encountered in clinical trials and reliability studies where right censoring is prevalent*.

### 3.2. Simulation Study

In this subsection, we carry out an extensive Monte Carlo simulation study to rigorously evaluate the finite-sample performance of the proposed test statistics defined in (7) and (10). The assessment is based on a systematic comparison of their empirical power functions against a broad class of alternative lifetime models that are widely used in economics, engineering, and reliability analysis.

To ensure a representative and practically relevant evaluation, we consider several parametric distribution families exhibiting distinct aging behaviors. Specifically, we examine the linear failure rate (LFR), also known as the linear exponential distribution, which includes the exponential and Rayleigh distributions as special cases and is frequently employed in reliability engineering and survival analysis. We also consider the Makeham distribution, whose additional parameter provides flexibility in modeling real-world lifetime data with non-constant hazard rates; this distribution is particularly relevant in actuarial science, demography, survival analysis, and reliability studies. Furthermore, the gamma distribution is included due to its widespread applications in econometrics, Bayesian statistics, and life-testing experiments; when the shape parameter is an integer, it reduces to the well-known Erlang distribution. Finally, the Weibull distribution, a cornerstone model in lifetime analysis, is incorporated to capture a wide range of failure mechanisms observed in engineering systems and natural phenomena. The specific parameterizations of all considered distributions are summarized in Table 1.

A key objective of the simulation study is to investigate the influence of the tuning parameters n and b on the behavior and power of the proposed tests. Thanks to the scale-invariance property of the test statistics, the empirical power can be evaluated without loss of generality by generating samples from the LFR, Makeham, gamma, and Weibull distributions under various parameter configurations, generically denoted by θ. This property allows for a meaningful comparison across different alternatives without the need for additional standardization.

Critical values for the estimators ${\hat{\delta }}_{n,b}^{1}$ and ${\hat{\delta }}_{n,b}^{2}$ are obtained through Monte Carlo simulation under the null hypothesis of exponentiality. Specifically, 10,000 independent samples of size N=25 are generated from the exponential distribution, and the empirical (1−α)-th quantiles of the resulting distributions of the estimators are used as critical values at significance level α. The choice N=25 is motivated by the goal of identifying suitable values of the tuning parameters n and b; similar qualitative conclusions were observed for other sample sizes and parameter settings. Denoting these critical values by ${\hat{\delta }}_{n,b}^{1}\left(1-\alpha \right)$ and ${\hat{\delta }}_{n,b}^{2}\left(1-\alpha \right)$, the null hypothesis is rejected at level α = 0.05 whenever ${\hat{\delta }}_{n,b}^{1}>{\hat{\delta }}_{n,b}^{1}\left(1-\alpha \right)$ and ${\hat{\delta }}_{n,b}^{2}>{\hat{\delta }}_{n,b}^{2}\left(1-\alpha \right).$

**Figure 1:**
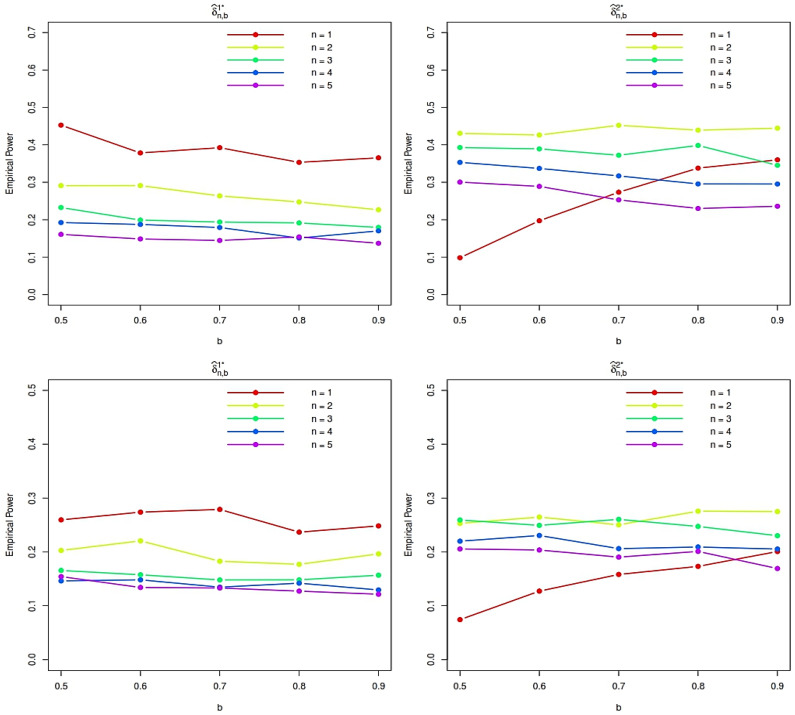
Empirical power function plots for LFR (**top**) and Makeham (**bottom**) distributions with N=25 and θ=1.5.

**Figure 2:**
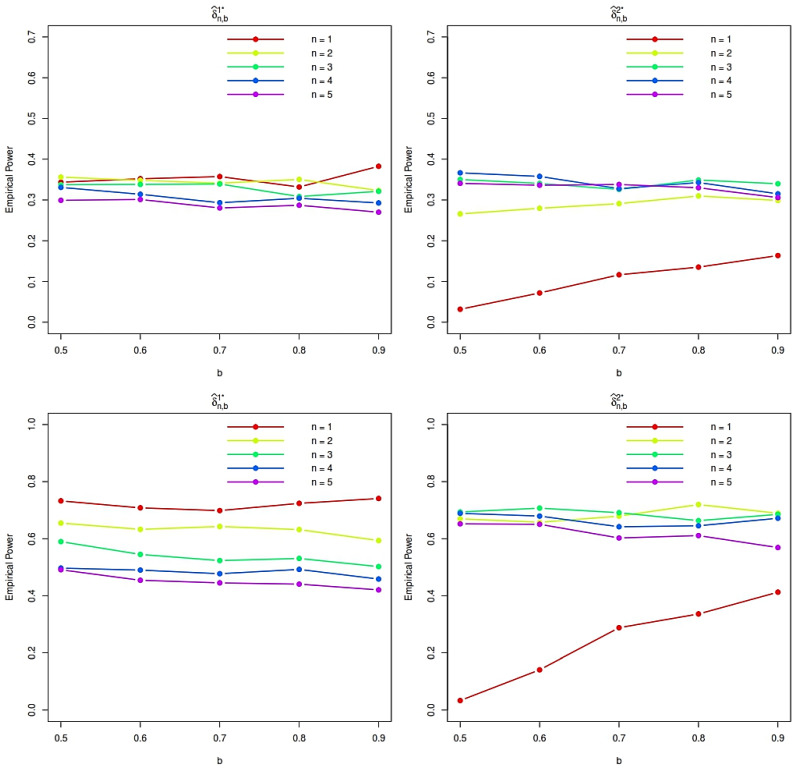
Empirical power function plots for Gamma (**top**) and Weibull (**bottom**) distributions with N=25 and θ=1.5.

The empirical power of the proposed tests is then estimated using 10,000 Monte Carlo replications under each alternative distribution. The resulting power curves, displayed in Figure 1 and Figure 2, provide detailed insight into the sensitivity of the proposed procedures to departures from exponentiality and clearly illustrate the impact of the tuning parameters *n* and *b*. These findings guide the selection of practically effective parameter values and highlight the robustness of the proposed tests across a wide range of alternatives. Finally, to further demonstrate the effectiveness of the proposed methodology, we compare its empirical power with that of several established tests for exponentiality against IFRA alternatives.

The following paragraphs present a structured overview of the competing tests considered in this study, outlining their underlying principles, test statistics, and asymptotic properties. This overview is provided to facilitate a clear and objective comparison between these existing procedures and the proposed test statistics ${\hat{\delta }}_{n,b}^{1}$ and ${\hat{\delta }}_{n,b}^{2}$, thereby highlighting the relative strengths and performance of the proposed methodology. Jayant and Deshpande [11] proposed the statistic $J_{a}$, defined as:

$$J_{a}=\frac{\sum_{i=1}^{N}\;R_{i}}{N(N-1)}-\frac{N+1}{2(N-1)}-\frac{1}{N-1},$$

where $R_{i}$ is the rank of $X_{i}$ in the combined sequence of $X_{1},\ldots ,X_{N}$ and $aX_{1},\ldots ,aX_{N}$ sorted in ascending order. The null hypothesis is rejected for large values of $J_{a}$. Aly [29] defined an empirical estimator $t_{1}(a,c)$ based on a measure of departure of the IFRA distribution from the exponential distribution:

$$t_{1}(a,c)=\frac{1}{N^{c+1}}\sum_{i=1}^{N}\;{\left(N+i-R_{i}\right)}^{c}$$

where a>1 and c>0 are fixed. The null hypothesis is rejected for large values of $t_{1}(a,c)$. Jammalamadaka et al. [30] developed a scale-invariant test statistic based on a moment inequality approach:

$$T_{N}=\frac{1}{N(N-1)}\sum_{i=1}^{N-1}\;\sum_{j=i+1}^{N}\;min\left(1,\frac{X_{i}}{X_{j}}\right).$$

The null hypothesis of exponentiality is rejected for large values of $T_{N}.$ A detailed power comparison of all tests under various alternative distributions and sample sizes is presented in Table 2, Table 3 and Table 4, which clearly illustrates the superiority of the proposed tests in most scenarios.

We evaluated the empirical power of the test statistics using 10,000 Monte Carlo replications for sample sizes *N* = 25, 50, and 100. Power was estimated as the proportion of replicates in which the test statistic exceeded its critical value (determined under the null hypothesis of exponentiality at the 5 percent significance level). As expected, the power of all tests increases with sample size, confirming their consistency. The test statistics ${\hat{\delta }}_{n,b}^{1⋆}$ and ${\hat{\delta }}_{n,b}^{2⋆}$ based on the specific values of n=1,2,4 and b=0.5,0.7,0.8, typically outperforms the competing tests including $J_{0.5},t_{1}(3,0.5),T_{N}$, and B for the alternatives, especially at moderate to large sample sizes. This highlights its high sensitivity to departures from exponentiality in these families. However, for the Weibull alternative with shape parameter θ>1 (which belongs to the IFRA class), ${\hat{\delta }}_{1,0.5}^{1⋆}$ shows relatively higher power compared to ${\hat{\delta }}_{n,b}^{2⋆}$ for n=2,4 and b=0.5,0.7,0.8, and some classical tests in small samples (e.g., N=25). In contrast, ${\hat{\delta }}_{n,b}^{1⋆}$ exhibits markedly improved performance as N grows, achieving near-perfect power for Weibull and Gamma alternatives when N=100. This suggests that ${\hat{\delta }}_{n,b}^{1⋆}$ is particularly well suited for detecting IFRA alternatives in larger samples. Overall, both proposed statistics are effective, but their relative performance depends on the underlying alternative and sample size.

Overall, the simulation results suggest that moderate choices of the tuning parameters *n* and *b* provide a favorable trade-off between sensitivity and stability across the considered alternatives. In particular, the statistic ${\hat{\delta }}_{n,b}^{1}$ tends to exhibit higher power for stronger IFRA departures and moderate sample sizes, whereas ${\hat{\delta }}_{n,b}^{2}$ performs competitively for smoother alternatives and larger samples. Based on these findings, we recommend using moderate values of *n* and *b* in practical applications, as they consistently yield robust performance without requiring fine parameter tuning.

### 3.3. Real Data Example

To illustrate the application of our test statistics in validating the IFRA property, we present numerical examples using real-life datasets from reliability contexts. Specifically, we apply the proposed tests, ${\hat{\delta }}_{n,b}^{1}$ and ${\hat{\delta }}_{n,b}^{2}$. Before testing for the IFRA class, statistical evidence suggesting the sample belongs to this class is required. We obtain such evidence using the TTT plot, a graphical method introduced by Barlow and Campo [31], commonly used in reliability analysis to investigate the IFR property (which implies IFRA). For a sample of ordered lifetimes $X_{1:N}\leq X_{2:N}\leq \cdots ,X_{N:N}$, let $D_{k}=(n-k+1)\left(X_{k:N}-X_{k-1:N}\right)$ for k=1,2,…,i, and $S_{i}=\sum_{k=1}^{i}\;D_{k}$ for i=1,…,N, with $S_{0}=0$, representing the total time on test at $X_{k:N}$. The TTT-plot is constructed by plotting $\left(\frac{i}{N},\frac{S_{i}}{S_{N}}\right)$ for i=1,…,N and connecting these points with straight lines. For the IFR aging class, the TTT plot from an IFR distribution is expected to be concave, while for an exponential distribution, it approximates the diagonal of the unit square. We employed this TTT plot technique as a preliminary diagnostic check for the sample datasets before formally testing for the aging class.

**Dataset 1.** *This dataset, reported in Proschan [32], contains* n=27 *observations representing the intervals between successive failures of air-conditioning systems in 7913 Boeing 720 jet airplanes. The observations are: 10, 14, 20, 23, 24, 25, 26, 29, 44, 44, 49, 56, 59, 60, 61, 62, 70, 76, 79, 84, 90, 101, 118, 130, 156, 186, 208, 208, 310*.

**Figure 3:**
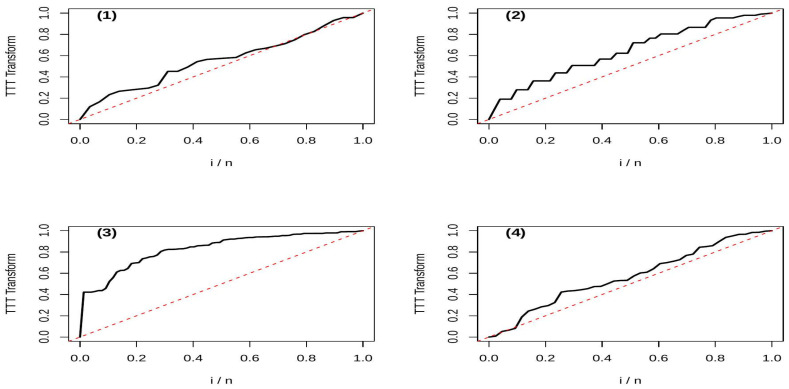
TTT-plots for datasets (1)–(4), where the solid lines denote the empirical TTT transforms and the dashed red lines indicate the exponential reference line.

*The TTT plot given in* Figure 3* number (1) shows a pattern that remains close to the diagonal, suggesting no strong evidence of aging IFR and hence IFRA. This visual impression is confirmed by the proposed test statistics, all of which yield p-values greater than 0.05 (see *Table 5*). Therefore, the null hypothesis of exponentiality is not rejected. This finding is consistent with a well-maintained system where failures occur randomly over time, without significant wear-out or deterioration, a typical scenario in reliability engineering when preventive maintenance is effective*.

**Dataset 2.** *This dataset, taken from Table 8.3.1 of Nelson [33], presents life test data for *n=51 *an old snubber design of a toaster component: 12, 17, 7, 13, 5, 2, 12, 2, 6, 4, 5, 14, 6, 2, 4, 18, 4, 19, 5, 14, 20, 8, 11, 26, 1, 3, 10, 18, 6, 10, 23, 7, 20, 4, 7, 6, 12, 10, 20, 3, 12, 3, 18, 18, 14, 14, 8, 6, 22, 11, 8*.

*The TTT plot given in* Figure 3* number (2) plot exhibits clear concavity, indicating an IFRA behavior. The proposed tests yield highly significant p-values providing strong evidence against exponentiality in favor of an IFRA alternative (see *Table 5*). This suggests that the component experiences deterioration over time, likely due to material fatigue or accumulated stress, a common aging pattern in mechanical systems*.

**Dataset 3.** *This dataset, from Thomas and Jose [34], consists of* n=77 *observations of geoelectrically derived parameters representing aquifer thickness: 10.49, 8.8, 12.42, 4.58, 6.85, 4.58, 5.0, 4.75, 4.75, 12.25, 9.5, 13.54, 10.42, 4.65, 9.88, 6.21, 8.6, 7.06, 7.96, 7.89, 9.7, 13.9, 12.65, 10.0, 12.65, 12.07, 9.8, 13.54, 9.82, 13.54, 12.42, 12.73, 12.22, 12.25, 12.32, 8.75, 12.0, 17.5, 11.88, 13.13, 13.56, 15.44, 13.22, 7.28, 11.7, 11.7, 11.6, 10.9, 11.84, 8.0, 10.2, 5.77, 13.9, 4.58, 12.07, 15.44, 10.2, 11.0, 8.5, 10.99, 10.39, 9.9, 13.94, 15.21, 13.56, 9.0, 20.47, 15.22, 11.5, 13.9, 13.22, 10.48, 15.48, 9.8, 12.21, 13.56, 7.04*.

*The TTT plot (*Figure 3* number (3)) is notably concave, and all proposed tests give p-values ≈ 0.0000, strongly supporting an IFRA model (see *Table 5*). This indicates that aquifer thinning follows an aging pattern, where the resource depletion rate increases over time, a meaningful finding in geological reliability and resource sustainability studies*.

**Dataset 4.** *This dataset, from Bryson and Siddiqui [35], consists of* n=43 *observations which are survival times, in days from diagnosis, of patients suffering from chronic granulooytic leukemia which is presented as follows: 7, 47, 58, 74, 177, 232, 273, 285, 317, 429, 440, 445, 455, 468, 495, 497, 532, 571, 579, 581, 650, 702, 715, 779, 881, 900, 930, 968, 1077, 1109, 1314, 1334, 1367, 1534, 1712, 1784, 1877, 1886, 2045, 2056, 2260, 2429, 250*.

*The TTT plot (*Figure 3* number (4)) is approximately linear, and the test statistics yield p-values > 0.05 (see *Table 5*). Hence, exponentiality is not rejected, implying a constant hazard over the observed period. This aligns with medical contexts where certain diseases exhibit roughly constant mortality risk during specific phases, such as the chronic phase of leukemia*.

The four examples illustrate how the proposed tests, supported by TTT plots, can effectively discriminate between exponential (no aging) and IFRA (aging) behaviors in real reliability and survival contexts. The coherent results between graphical and formal testing procedures validate the practical utility of the methodology. The tests are particularly effective in moderate samples offering a robust nonparametric tool for reliability analysts, engineers, and medical researchers. These conclusions are in close agreement with the graphical patterns observed in the corresponding TTT plots, thereby providing coherent and mutually reinforcing empirical evidence.

## 4. Conclusions

In this paper, we developed two new nonparametric test statistics for assessing exponentiality against IFRA alternatives, constructed using the cumulative residual extropy and cumulative past extropy of the first-order statistic. Under mild regularity conditions, asymptotic normality of the proposed estimators was established, and scale-invariant versions were derived to ensure robustness with respect to an unknown scale parameter. A comprehensive Monte Carlo simulation study was conducted to evaluate finite-sample performance under several practically relevant alternatives, including linear failure rate, Makeham, gamma, and Weibull distributions. The results demonstrated that the proposed procedures exhibit competitive and often superior empirical power compared with several well-established tests, particularly for moderate to large sample sizes. The practical applicability of the methodology was further illustrated through real-data analyses, with conclusions supported by preliminary graphical diagnostics based on TTT plots. Several important extensions and open problems naturally arise from the present work. From a theoretical perspective, it would be of considerable interest to extend the proposed extropy-based testing framework to other fundamental aging classes, such as IFR and NBU, as well as their dual counterparts, and to derive the corresponding inequality structures that characterize departures from exponentiality. Another important direction concerns the optimal selection of the tuning parameters (n, b); developing theoretical guidelines based on asymptotic local power analysis or efficiency considerations would provide deeper insight into the operating characteristics of the proposed tests. From a methodological standpoint, the use of bootstrap or permutation-based calibration schemes may further enhance finite-sample accuracy, especially in small or moderate sample sizes where asymptotic approximations may be less reliable. Finally, extending the proposed methodology to censored and incomplete lifetime data, including right-censored and progressively censored samples commonly encountered in reliability and survival analysis, represents a promising avenue for future research.

## Figures and Tables

**Table 1:** Cumulative distribution function with the scale parameter θ.

Distribution	Cumulative Distribution Function	Support
**LFR**	$F_{1}(x)=1-e^{-\left(x+0.5\theta x^{2}\right)}$,	x>0,θ>0
**Makeham**	$F_{2}(x)=1-e^{-\left(x+\theta \left(x+e^{-x}-1\right)\right)}$,	x>0,θ>0
**Gamma**	$F_{3}(x)=1-\int_{0}^{x}\;\frac{t^{\theta -1}}{\Gamma (\theta )}e^{-t}dt$	x>0,θ>0
**Weibull**	$F_{4}(x)=1-e^{-x^{\theta }}$,	x>0,θ>0

**Table 2:** Power comparisons of the tests in significance level α=0.05 and N=25.

N	θ	$J_{0.5}$	*t*_1_ (3, 0.5)	$T_{N}$	${\hat{\delta }}_{1,0.5}^{1^{⋆}}$	${\hat{\delta }}_{1,0.7}^{1^{⋆}}$	${\hat{\delta }}_{1,0.8}^{1^{⋆}}$	${\hat{\delta }}_{2,0.7}^{2^{⋆}}$	${\hat{\delta }}_{2,0.8}^{2^{⋆}}$	${\hat{\delta }}_{4,0.5}^{2^{⋆}}$
LFR	1.2	0.2921	0.2582	0.2903	0.3854	0.3449	0.3175	0.3883	0.3848	0.2948
	1.5	0.3475	0.3034	0.3412	0.4378	0.3847	0.3714	0.4471	0.4474	0.3497
	2.0	0.4036	0.3565	0.3956	0.5174	0.4586	0.4419	0.5036	0.5042	0.4060
	2.5	0.4541	0.3964	0.4470	0.5682	0.4963	0.4961	0.5603	0.5454	0.4662
	3.0	0.4921	0.4317	0.4883	0.6161	0.5340	0.5295	0.5881	0.6099	0.4956
Makeham	1.2	0.1947	0.1865	0.1946	0.2296	0.2044	0.2210	0.2131	0.2266	0.1988
	1.5	0.2270	0.2185	0.2381	0.2677	0.2489	0.2499	0.2731	0.2541	0.2375
	2.0	0.2758	0.2697	0.2896	0.3323	0.3063	0.3157	0.3033	0.3220	0.2977
	2.5	0.3193	0.2977	0.3350	0.3820	0.3541	0.3622	0.3704	0.3807	0.3316
	3.0	0.3596	0.3352	0.3720	0.4420	0.3860	0.3875	0.4240	0.4057	0.3734
Gamma	1.2	0.1490	0.1327	0.1396	0.1397	0.1461	0.1385	0.1305	0.1372	0.1464
	1.5	0.3681	0.3220	0.3688	0.3566	0.3554	0.3417	0.2948	0.3030	0.3466
	2.0	0.7577	0.6627	0.7560	0.7236	0.7350	0.7565	0.6048	0.6414	0.7136
	2.5	0.9344	0.8603	0.9390	0.9237	0.9333	0.9358	0.8434	0.8560	0.9253
	3.0	0.9881	0.9526	0.9894	0.9799	0.9841	0.9857	0.9486	0.9501	0.9796
Weibull	1.2	0.2399	0.2111	0.2430	0.2476	0.2579	0.2335	0.2262	0.2247	0.2270
	1.5	0.7061	0.6026	0.7172	0.7326	0.7089	0.7268	0.6715	0.6844	0.6823
	2.0	0.9868	0.9555	0.9919	0.9960	0.9920	0.9909	0.9897	0.9902	0.9833
	2.5	0.9998	0.9985	0.9999	1.0000	1.0000	1.0000	0.9999	0.9998	1.0000
	3.0	0.9999	1.0000	1.0000	1.0000	1.0000	1.0000	1.0000	1.0000	1.0000

**Table 3:** Power comparisons of the tests in significance level α=0.05 and N=50.

N	θ	$J_{0.5}$	*t*_1_(3, 0.5)	$T_{N}$	${\hat{\delta }}_{1,0.5}^{1^{⋆}}$	${\hat{\delta }}_{1,0.7}^{1^{⋆}}$	${\hat{\delta }}_{1,0.8}^{1^{⋆}}$	${\hat{\delta }}_{2,0.7}^{2^{⋆}}$	${\hat{\delta }}_{2,0.8}^{2^{⋆}}$	${\hat{\delta }}_{4,0.5}^{2^{⋆}}$
LFR	1.2	0.5010	0.4376	0.5007	0.6673	0.5704	0.5594	0.6384	0.6384	0.4729
	1.5	0.5664	0.5056	0.5620	0.7206	0.6582	0.6392	0.7190	0.6992	0.5548
	2.0	0.6571	0.5790	0.6565	0.8161	0.7458	0.7213	0.8069	0.7971	0.6435
	2.5	0.7350	0.6486	0.7238	0.8682	0.8013	0.7883	0.8572	0.8474	0.6962
	3.0	0.7601	0.6962	0.7700	0.9008	0.8472	0.8209	0.8846	0.8760	0.7574
Makeham	1.2	0.3028	0.2902	0.3209	0.3780	0.3630	0.3436	0.3808	0.3728	0.3279
	1.5	0.3781	0.3455	0.3933	0.4677	0.4438	0.4106	0.4533	0.4341	0.3810
	2.0	0.4683	0.4242	0.4812	0.5852	0.5551	0.5253	0.5660	0.5618	0.4736
	2.5	0.5402	0.4912	0.5581	0.6485	0.6139	0.6052	0.6293	0.6367	0.5613
	3.0	0.5994	0.5576	0.6224	0.7295	0.6752	0.6674	0.6889	0.7083	0.6141
Gamma	1.2	0.2099	0.1873	0.2338	0.1946	0.2147	0.2082	0.1731	0.1859	0.2039
	1.5	0.6263	0.5159	0.6560	0.5914	0.6167	0.6072	0.5112	0.5007	0.5957
	2.0	0.9679	0.9031	0.9767	0.9546	0.9645	0.9647	0.9020	0.9059	0.9511
	2.5	0.9996	0.9918	0.9995	0.9982	0.9991	0.9993	0.9896	0.9914	0.9967
	3.0	1.0000	0.9989	1.0000	1.0000	1.0000	1.0000	0.9991	0.9999	1.0000
Weibull	1.2	0.3978	0.3234	0.4173	0.4200	0.4252	0.3995	0.3895	0.4002	0.3803
	1.5	0.9367	0.8691	0.9579	0.9629	0.9548	0.9512	0.9424	0.9421	0.9245
	2.0	1.0000	0.9988	1.0000	1.0000	1.0000	1.0000	1.0000	1.0000	1.0000
	2.5	1.0000	1.0000	1.0000	1.0000	1.0000	1.0000	1.0000	1.0000	1.0000
	3.0	1.0000	1.0000	1.0000	1.0000	1.0000	1.0000	1.0000	1.0000	1.0000

**Table 4:** Power comparisons of the tests in significance level α=0.05 and N=100.

N	θ	$J_{0.5}$	*t*_1_(3, 0.5)	$T_{N}$	${\hat{\delta }}_{1,0.5}^{1^{⋆}}$	${\hat{\delta }}_{1,0.7}^{1^{⋆}}$	${\hat{\delta }}_{1,0.8}^{1^{⋆}}$	${\hat{\delta }}_{2,0.7}^{2^{⋆}}$	${\hat{\delta }}_{2,0.8}^{2^{⋆}}$	${\hat{\delta }}_{4,0.5}^{2^{⋆}}$
LFR	1.2	0.7422	0.6810	0.7576	0.9199	0.8542	0.8447	0.9055	0.8884	0.7302
	1.5	0.8246	0.7608	0.8248	0.9515	0.9108	0.8961	0.9467	0.9374	0.8028
	2.0	0.8891	0.8435	0.9054	0.9832	0.9624	0.9448	0.9758	0.9768	0.8758
	2.5	0.9321	0.8909	0.9418	0.9901	0.9776	0.9691	0.9901	0.9852	0.9254
	3.0	0.9564	0.9267	0.9603	0.9962	0.9892	0.9839	0.9938	0.9922	0.9425
Makeham	1.2	0.5367	0.4489	0.5277	0.6427	0.5977	0.5751	0.6202	0.6132	0.5082
	1.5	0.6249	0.5418	0.6160	0.7588	0.6986	0.6867	0.7259	0.7094	0.6103
	2.0	0.7455	0.6513	0.7417	0.8530	0.8081	0.7997	0.8313	0.8274	0.7113
	2.5	0.8344	0.7414	0.8178	0.9186	0.8914	0.8729	0.8920	0.8989	0.8064
	3.0	0.8823	0.8012	0.8776	0.9456	0.9273	0.9167	0.9351	0.9296	0.8527
Gamma	1.2	0.3444	0.2979	0.3612	0.3166	0.3347	0.3361	0.2965	0.2969	0.3220
	1.5	0.8945	0.7949	0.9040	0.8559	0.8761	0.8784	0.7760	0.7921	0.8569
	2.0	00.9998	0.9964	0.9997	0.9993	0.9996	0.9998	0.9948	0.9963	0.9992
	2.5	1.0000	1.0000	1.0000	1.0000	1.0000	1.0000	1.0000	1.0000	1.0000
	3.0	1.0000	1.0000	1.0000	1.0000	1.0000	1.0000	1.0000	1.0000	1.0000
Weibull	1.2	0.6555	0.5605	0.6665	0.7033	0.6786	0.6620	0.6404	0.6210	0.5986
	1.5	0.9988	0.9925	0.9994	0.9996	0.9991	0.9997	0.9989	0.9989	0.9970
	2.0	1.0000	1.0000	1.0000	1.0000	1.0000	1.0000	1.0000	1.0000	1.0000
	2.5	1.0000	1.0000	1.0000	1.0000	1.0000	1.0000	1.0000	1.0000	1.0000
	3.0	1.0000	1.0000	1.0000	1.0000	1.0000	1.0000	1.0000	1.0000	1.0000

**Table 5:** Statistical Test Results for Real Dataset.

Test	Dataset 1		Dataset 2		Dataset 3		Dataset 4	
	Statistic	***p***-Value	Statistic	***p***-Value	Statistic	***p***-Value	Statistic	***p***-Value
$J_{0.5}$	0.6983	0.1199	0.7422	0.0011	0.9144	0.0000	0.6977	0.0794
***t*_1_** **(3, 0.5)**	0.8945	0.0079	0.8666	0.0011	0.9798	0.0000	0.8820	0.0323
$T_{N}$	0.7289	0.0499	0.7516	0.0004	0.8603	0.0000	0.7160	0.0997
${\hat{\delta }}_{1,0.5}^{1^{⋆}}$	0.0198	0.1388	0.0659	0.0001	0.1609	0.0000	0.0300	0.0968
${\hat{\delta }}_{1,0.7}^{1^{⋆}}$	0.0238	0.0930	0.0385	0.0003	0.0947	0.0000	0.0193	0.0930
${\hat{\delta }}_{1,0.8}^{1^{⋆}}$	0.0147	0.0875	0.0239	0.0007	0.0617	0.0000	0.0114	0.0940
${\hat{\delta }}_{2,0.7}^{2^{⋆}}$	0.0225	0.2293	0.0546	0.0005	0.1172	0.0000	0.0297	0.0711
${\hat{\delta }}_{2,0.8}^{2^{⋆}}$	0.0133	0.2000	0.0334	0.0008	0.0765	0.0000	0.0170	0.0733
${\hat{\delta }}_{4,0.5}^{2⋆}$	0.0336	0.0731	0.0539	0.0023	0.1608	0.0000	0.0266	0.0745

## Data Availability

The original contributions presented in this study are included in the article. Further inquiries can be directed to the corresponding author.

## Abbreviations

*X*	Absolutely continuous random variable
*F(x)*	Cumulative distribution function (cdf) of (X)
$\bar{F}(x)$	Survival function of (X)
*f(x)*	Probability density function (pdf) of (X)
H(X)	Shannon differential entropy
$J\left(X\right)$	Differential extropy
$EJ\left(X\right)$	Cumulative residual extropy (CREX)
$\bar{ℇ}J\left(X\right)$	Cumulative past extropy (CPEX)
$X_{1:n}$	First-order statistic of a sample of size *n*
$\delta_{n,b}^{1}$	Test statistic based on CREX of $X_{1:n}$
$\delta_{n,b}^{2}$	Test statistic based on CPEX of $X_{1:n}$
IFRA	Increasing failure rate average
TTT	total time on test
